# Antifungal Activity of *N*-(4-Halobenzyl)amides against *Candida* spp. and Molecular Modeling Studies

**DOI:** 10.3390/ijms23010419

**Published:** 2021-12-31

**Authors:** Yunierkis Perez-Castillo, Ricardo Carneiro Montes, Cecília Rocha da Silva, João Batista de Andrade Neto, Celidarque da Silva Dias, Allana Brunna Sucupira Duarte, Hélio Vitoriano Nobre Júnior, Damião Pergentino de Sousa

**Affiliations:** 1 Bio-Cheminformatics Research Group and Escuela de Ciencias Físicas y Matemáticas, Universidad de Las Américas, Quito 170504, Ecuador; yunierkis@gmail.com; 2 Department of Pharmaceutical Sciences, Federal University of Paraíba, João Pessoa 58051-900, PB, Brazil; ricsony_79@yahoo.com.br (R.C.M.); celidarque@ltf.ufpb.br (C.d.S.D.); allanabrunna@gmail.com (A.B.S.D.); 3 Laboratory of Bioprospection in Antimicrobial Molecules (LABIMAN), Drug Research and Development Center (NPDM), Federal University of Ceará, Fortaleza 60020-181, CE, Brazil; ceciliarocha86@yahoo.com.br (C.R.d.S.); jb_andradeufc@hotmail.com (J.B.d.A.N.); helioufc@yahoo.com.br (H.V.N.J.)

**Keywords:** antimicrobial activity, *Candida auris*, benzoic acid, fungi, cinnamic acid, natural products, plants, molecular docking, candidiasis, anticandidal drugs

## Abstract

Fungal infections remain a high-incidence worldwide health problem that is aggravated by limited therapeutic options and the emergence of drug-resistant strains. Cinnamic and benzoic acid amides have previously shown bioactivity against different species belonging to the *Candida* genus. Here, 20 cinnamic and benzoic acid amides were synthesized and tested for inhibition of *C. krusei* ATCC 14243 and *C. parapsilosis* ATCC 22019. Five compounds inhibited the *Candida* strains tested, with compound **16** (MIC = 7.8 µg/mL) producing stronger antifungal activity than fluconazole (MIC = 16 µg/mL) against *C. krusei* ATCC 14243. It was also tested against eight *Candida* strains, including five clinical strains resistant to fluconazole, and showed an inhibitory effect against all strains tested (MIC = 85.3–341.3 µg/mL). The MIC value against *C. krusei* ATCC 6258 was 85.3 mcg/mL, while against *C. krusei* ATCC 14243, it was 10.9 times smaller. This strain had greater sensitivity to the antifungal action of compound **16**. The inhibition of *C. krusei* ATCC 14243 and *C. parapsilosis* ATCC 22019 was also achieved by compounds **2**, **9**, **12**, **14** and **15**. Computational experiments combining target fishing, molecular docking and molecular dynamics simulations were performed to study the potential mechanism of action of compound **16** against *C. krusei*. From these, a multi-target mechanism of action is proposed for this compound that involves proteins related to critical cellular processes such as the redox balance, kinases-mediated signaling, protein folding and cell wall synthesis. The modeling results might guide future experiments focusing on the wet-lab investigation of the mechanism of action of this series of compounds, as well as on the optimization of their inhibitory potency.

## 1. Introduction

The yeasts of genus *Candida* cause several infectious diseases such as candidiasis [1] and candidemia [2]. These involve a wide spectrum of superficial and invasive opportunistic mycoses affecting patients exposed to a great diversity of risk factors [3], such as deficiency in host defense mechanisms, invasive medical procedures and impairment of anatomical barriers due to burns [4]. In the search for chemical agents that could defeat these endemics, benzoic acids and their derivatives have a peculiar power to inhibit the growth of some pathogenic fungi [5,6]. The inhibition of benzoate 4-hydroxylase, an enzyme responsible for aromatic detoxification, is a known route for benzoic microbial inhibition [7]. Benzoic acids and their derivatives, such as gallic acid, an anti-inflammatory and neuroprotective agent [8,9,10,11]; vanillic acid, which functions as a hepatoprotective agent [12] and cinnamaldehyde, a phenylpropanoid that has antimicrobial properties on *Staphylococcus aureus* strains as well as on gram-negative bacteria, are important therapeutic sources [13].

The occurrence of halogenated cinnamic and benzoic amides is not usual in plants, but research has been looking up for applied synthesis of these halogenated structures [14,15,16,17,18], which show important biological activities such as antitumoral properties, EGFR inhibition [19], stimulatory effects on insulin secretion [20] and melanogenesis inhibition [12]. Some halogenated amides have a high inhibitory antimicrobial activity, and it was proven that chlorinated cinnamic analogues possess higher microbial inhibition in bacterial and fungal strains than their fluorinated and brominated analogues [21]. In addition, benzoic derivatives of the type salicylanilides with *ortho* and *para* hydroxyl and *meta* methoxyl groups have increased microbial inhibitory activity [22].

On the other hand, computational methods have been widely applied for the prediction of potential targets of bioactive compounds. These are based on the information available in the literature and databases containing protein–ligand associations, employing either ligand- or structure-based methodologies [23,24,25,26,27]. Experimental evidence supporting the binding of compounds of interest to their computationally predicted targets has been provided elsewhere [28,29,30]. Furthermore, as computational power constantly increases, the large-scale post-processing of predicted ligand–target associations employing computationally demanding methods, such as molecular dynamics simulations, is becoming a routine task [31,32,33].

In previous works [34,35], we performed the preparation of halogenated cinnamic and benzoic amides with benzotriazol-1-yloxy-tris[dimethylamino]phosphonium hexafluorophosphate (BOP) as a coupling agent [36,37]. In this work, we continue the prospection of their antifungal activity by evaluating the inhibitory capacity of halogenated amides in other strains of the *Candida* genus. The antifungal activity evaluations are followed by computational predictions to determine the possible mechanisms of action of the most potent compound in our experimental assays.

## 2. Results

### 2.1. Chemistry

**Scheme 1:**
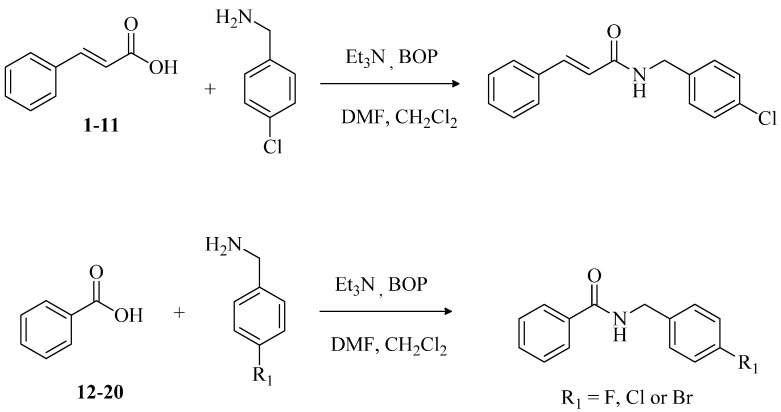
General procedure for synthesis of halogenated amides.

The structures of amides **1**–**20** were consistent with the infrared spectra (IR), ^1^H- and ^13^C-NMR and high-resolution mass spectra (MALDI) data, according to previous work [34]. The estimated purity values of the amides were at 92–96% by ^1^H-NMR [38]. Products **1**–**11** are 4-chlorobenzylamides with cinnamic skeletal structure, and compounds **12**–2**0** are *N*-(4-halobenzyl)amides with benzoic skeletal structure. The synthesis of the products was conducted by the same procedures as those in Scheme 1.

### 2.2. Antimicrobial Activity

In the antifungal study, the samples tested on the fungal strains *C. parapsilosis* (ATCC 22019) and *C. krusei* (ATCC 14243) were analyzed, and the results are shown in Table 1. The MICs of the compounds were significantly different, ranging from 250 to 7.8 µg/mL. The MIC is the representation of the geometric mean of two distinct experiments on two different days. According to Table 1, the relevant MICs in the inhibition of *C. parapsilosis* were achieved by compounds **15** and **9** with 250 µg/mL, compounds **12** and **16** with 150 µg/mL and compounds **2** and **14** with 31.25 µg/mL, while in the inhibition of *C. krusei*, an MIC of 250 µg/mL was obtained for compounds **2**, **12** and **15**, an MIC of 150 µg/mL for **9**, an MIC of 31.25 µg/mL for **14** and an MIC of 7.8 µg/mL for **16**.

### 2.3. Antifungal Activity of Compound **16**

The amide **16** was selected to test against several Candida strains, including five clinical strains resistant to fluconazole (Table 2). The compound **16** exhibits antimicrobial activity against all strains tested, with MICs ranging from 85.3 to 341.3 µg/mL.

### 2.4. Molecular Modeling

The list of potential targets for compound **16** in *C. krusei*, selected as described in the Material and Methods section, is provided in Table 3. From the 37 selected proteins, 17 were identified through the computational target fishing approach, while the remaining 20 belong to the ergosterol synthesis pathway. Homology models were generated for all the proteins. The templates used in the homology models, the coverage of the query sequences by the templates and the values of the QMEAN scores of the models are provided as Supplementary Materials in Table S1. Among all targets, the models for seven of them (ERG6, ERG26, ERG4, ERG5, ERG3, ERG25 and ERG24) were discarded due to their low quality (QMEAN < −4).

Compound **16** was docked to the 30 proteins for which valid homology models could be generated, and the results of this step are presented as Supplementary Materials in Table S2. No docking solution could be found for ERG1; thus, 29 possible targets for the compound were further analyzed. The complexes predicted for these 29 proteins had the ligand within the binding cavity, showing interactions with functionally relevant residues. In addition, for seven out of twenty-nine proteins, only one valid pose of compound **16** was identified, while more than one ligand conformer was selected for the rest of the targets. In total, 67 potential ligand–receptor complexes were selected for additional analyses.

Molecular docking algorithms are designed for processing large amounts of compounds in a reasonable time. This processing speed comes at the cost of either neglecting or simplifying several factors involved in molecular recognition. Because of this, it is recommended that the post-processing of docking predicted complexes employ more accurate modeling methods. In this sense, the estimation of the free energies of binding from MD simulations had been proposed as a strategy for the refinement of the results obtained with molecular docking [31,39]. Hence, instead of analyzing the most probable targets of compound **16** from the results of the molecular docking calculations, MD simulations were performed and the free energies of binding were predicted, as described in the Materials and Methods section.

**Figure 1:**
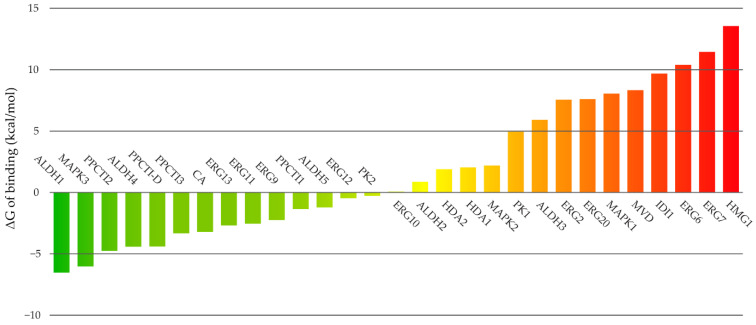
Predicted free energies of binding of compound 16 to its potential targets.

The simulations lead to 1.34 µs of total simulation time across all predicted complexes, and the most stable conformation of compound **16** to each target was selected as that with the lowest value of predicted ΔG of binding. The detailed results of the MM-PBSA calculations are provided as Supplementary Materials in Table S3 and summarized in Figure 1.

According to the predicted free energies of binding, the most probable targets of compound **16** in *C. krusei* are ALDH1, MAPK3, PPCTI2, ALDH4 and PPCTI-D. Previous studies showed that amides derived from vanillic acid (highly similar to the compounds studied herein) interfere with the synthesis of ergosterol in *C. guilliermondii* [40]. Among the targets belonging to the ergosterol synthesis pathway, the firsts in the ranked list are ERG13, ERG11 and ERG9 at positions 8, 9 and 10, respectively. Despite these positions, negative values of ΔG of binding are predicted for the later proteins, which indicates the possibility of binding the ligand to them.

**Figure 2:**
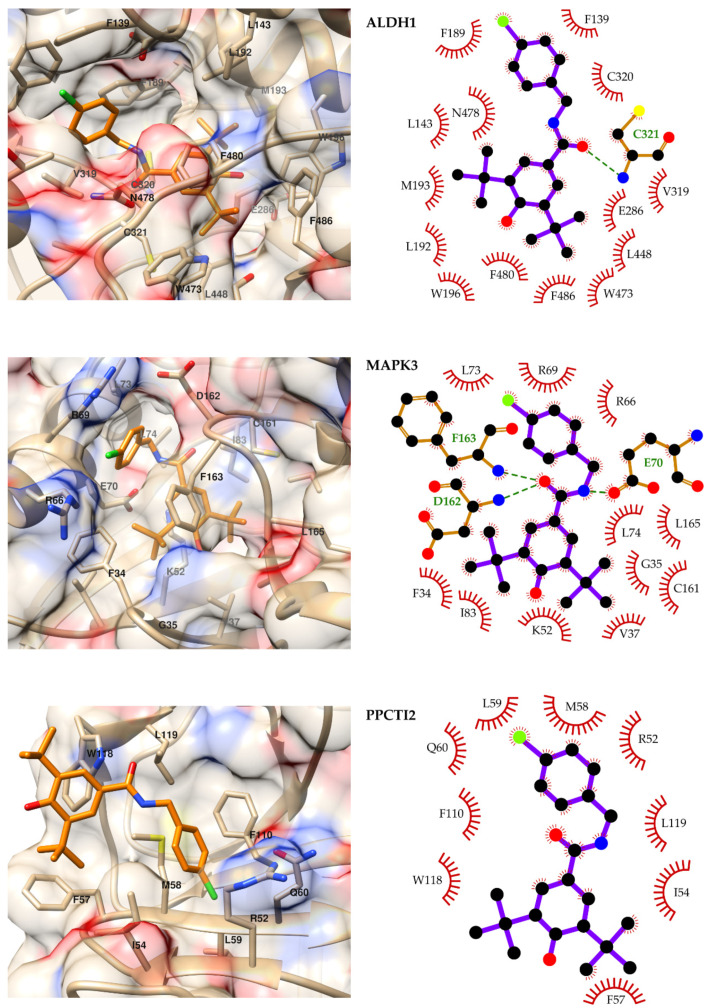
Predicted binding modes of compound **16** to ALDH1, MAPK3 and PPCTI2 (**left**) and diagrams of the predicted ligand–receptor interactions (**right**). For representing the predicted binding modes, compound **16** is depicted in orange, and the following scheme is used for non-carbon atoms: oxygen is red, nitrogen blue, chlorine green and sulfur yellow. In the interaction diagrams (**left**), carbon atoms are depicted in black, and heavy atoms are represented only for residues forming hydrogen bonds with the ligand.

Based on the modeling results, the binding modes of compound **16** to the top three target candidates ALDH1, MAPK3 and PPCTI2, as well as to the best-ranked proteins from the ergosterol synthesis pathway (ERG13, ERG11 and ERG9), were analyzed in detail. Figure 2 summarizes the predicted binding mode of the ligand to ALDH1, MAPK3 and PPCTI2, as well as the diagrams of the compound **16**–receptor interactions. To select the structure for representation purposes, the 100 MD snapshots used for MM-PBSA calculations were clustered, and the centroid of the most populated cluster was chosen as the representative structure. Only receptor residues interacting with the ligand in more than 50% of the analyzed snapshots are labeled and represented in the diagrams of interactions. The figures of the complexes were produced with UCSF Chimera [41], the frequencies of ligand–receptor interactions were analyzed with the aforementioned software and Cytoscape [42] and the interaction diagrams were obtained with Ligplot+ [43].

Compound **16** is predicted to bind to ALDH1 by blocking the access of the substrates to the catalytic residues and the cofactor. Its di-tert-butylphenol moiety orientates toward the bottom of the binding cavity, a mostly hydrophobic region lined by L143, L192, M193, W196, E286, L448, W473, F480 and F486. In addition, this substituent is favorably placed for π–π stacking with F480. The carbonyl oxygen is predicted to accept a hydrogen bond from the side chain amine of C321 in 86% of the analyzed MD snapshots. The amide nitrogen of compound **16** is oriented to interact with N478, and it is observed to occasionally (16% of the snapshots) donate a hydrogen bond to the side chain of this residue. Finally, the chloro-benzene substituent occupies the entrance of the binding cavity, mainly interacting with F139, F189 and V319.

In the predicted complex with MAPK3, the amide nitrogen of compound 16 serves as a hydrogen bond donor to the side chain of E70, while the carbonyl oxygen accepts hydrogen bonds from the backbones of D162 and F163. The halogenated benzene ring occupies the entrance of the cavity, interacting with F34, R66, R69, L73 and D162. The other side of the compound is buried within the enzyme’s active site and interacts with V37, K52, I83, C161, L165 and F163. This ring also positions favorably for stacking in front of F163.

The potential target of compound **16** ranked in the third position is PPCTI2. In this case, the ligand is predicted to bind at one end of the large substrate-binding groove. The di-tert-butylphenol substituent is predicted to be exposed to the solvent, with one of the tert-butyl moieties interacting with I54 and F57 and the aromatic ring contacting W118. The central linker of the compound makes contact with M58, W118 and L119, while the chlorine atom occupies a sub-pocket formed by R52, I54, M58, L59 and Q60. Additionally, the ligand’s halogenated ring is located between I54, M58 and F110, with the possibility of stacking perpendicularly to the latter residue.

**Figure 3:**
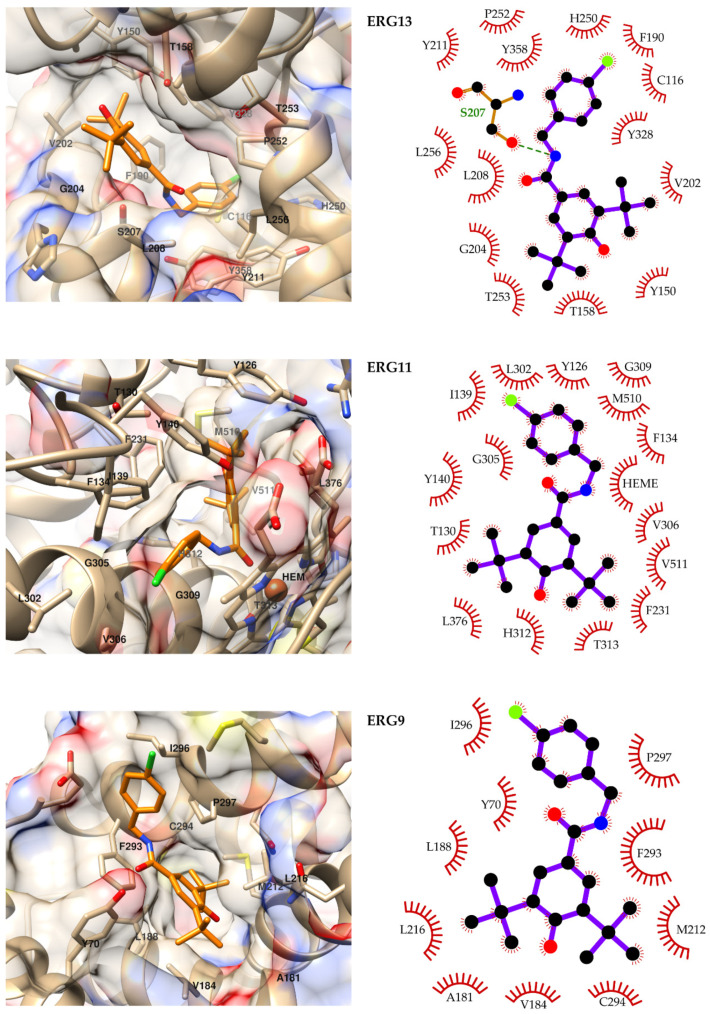
Predicted binding modes of compound 16 to ERG13, ERG11 and ERG9 (**left**) and diagrams of the predicted ligand–receptor interactions (**right**). For representing the predicted binding modes, the ligand is depicted in orange, and the following scheme is used for non-carbon atoms: oxygen is red, nitrogen blue, chlorine green and sulfur yellow. In the interaction diagrams (**left**), carbon atoms are depicted in black, and heavy atoms are represented only for residues forming hydrogen bonds with the ligand.

In Figure 3 are presented the predicted conformations of compound **16** bound to the ergosterol synthesis pathway enzymes ERG13, ERG11 and ERG9. The predicted ligand–ERG13 complex shows the compound filling the binding site of the CoA derivative substrates, with the chlorophenyl substituent occupying the bottom of it. The chlorine atom makes contact with C116, Y238 and H250, while the aromatic ring is accommodated in a hydrophobic region defined by Y211, P252, L256 and Y358. One hydrogen bond between the amide nitrogen of the ligand and the side chain of S207 stabilizes the complex in 55% of the analyzed MD snapshots. In addition, the di-tert-butylphenol substituent is placed at the mouth of the cavity and interacts with Y150, T158, V202 and G204.

The model obtained for ERG11 predicts the halogenated ring and the central linker of the compound facing the heme cofactor, with the carbonyl atom of the former contacting the Fe^2+^ ion of the latter. This aromatic moiety also interacts with F134, I139, G305, V306 and G309. The rest of the molecule forms a large network of interactions with Y126, T130, Y140, F231, H312, T313, L376, M510 and V511. Finally, the predicted complex of compound 16 with ERG9 is predicted to stabilize through Van der Waals and hydrophobic interactions. In this complex, the ligand’s di-tert-butylphenol group occupies the bottom of the binding pocket and mainly interacts with M212, L216, F293 and C294. The central linker of the compound makes contact with Y70 and F293, while the halogenated ring is exposed to the solvent and only interacts with I296 and P297.

**Figure 4:**
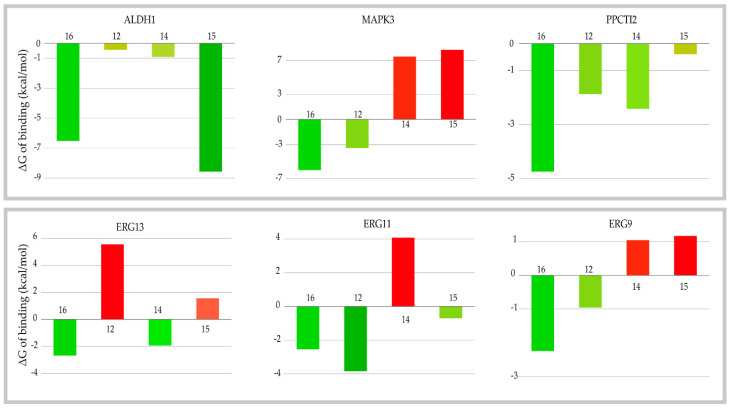
Predicted free energies of binding of compounds **12**, **14** and **15** to ALDH1, MAPK3, PPCTI2, ERG13, ERG11 and ERG9.

To get additional insight into the potential antifungal mechanism of action of this series of compounds and the influence of the substituents at positions R2, R3 and R4 (see Table 1), compounds **12**, **14** and **15** were subject to the same modeling strategy applied to **16**. The predicted free energies of binding of these compounds to the ALDH1, MAPK3, PPCTI2, ERG13, ERG11 and ERG9 proteins are summarized in Figure 4 and provided as Supplementary Materials in Table S4. The results of these calculations show that compound **16** is the only one predicted with negative free energy of binding for all targets. Interestingly, compound **15** shows better a inhibition profile against ALDH1 than **16**, while the same behavior is predicted for **12** and ERG11.

The representative complexes predicted for compounds **12**, **14**, **15** and **16** with the most probable targets of the latter were analyzed in detail to better understand the results presented in Figure 4. All these complexes are provided in PDB format as Supplementary Materials in the file RepComplexes.zip. The improved free energy of binding of **15** relative to **16** in the complex with ALDH1 can be explained by the lack of tert-butyl moieties in the former. The lack of these groups makes the phenol substituent less bulky and allows compound **15** to occupy a deeper region of the binding pocket without losing the predicted hydrogen bond with C321. This movement positions the phenol group favorably for additional hydrogen bonding to the side chain of E286, thus increasing the stability of the ALDH1–**15** complex. On the other hand, the absence of the tert-butyl and hydroxy substituents in **12** reduces the stability of the complex of this compound with ALDH1. For the predicted ALDH1–**14** complex, the reduction in hydrophobicity and volume compared to compound **16,** produced by the introduction of the methoxy substituents, might be responsible for the increase in the free energy of binding for this complex.

Despite the highly similar orientations of the four ligands in the complexes with MAPK3, there are large differences in their predicted free energies of binding. These can be explained by considering the energy components (see Table S4). The lowest Van der Waals component for compound 16 is related to its ability to better complement the receptor by pointing the bulky tert-butyl groups to sub-pockets delimited on each side by V54 and L151. In addition, the lack of a hydroxy group in compound **12** leads to a reduction (improvement) in the polar solvation energy component, suggesting that the presence of this group is unfavorable for ligand binding to MAPK3.

For the PPCTI2 target, the chloro-benzene moiety occupies the interior of the binding cavity while the substituted phenol ring is located at the entrance of the pocket, partially exposed to the solvent. This orientation of the compounds correlates with the fact that the main differences in the predicted free energies of binding are related to the Van der Waals contribution. For this reason, the substituted rings of compounds **14** and **16** are favored in terms of Van der Waals interactions and hence of total free energy of binding. This binding pattern with the chloro-benzene group occupying the bottom of the binding pocket is also observed for ERG13. In this case, ligand binding is predicted as being mainly driven by Van der Waals interactions as well, which favors the free energy of bonding of compounds **14** and **16**. The worst performance in terms of binding energy to ERG13 is observed for compound **12**, which is also affected by an increased electrostatic energy component due to the reduction of frequency of hydrogen bonding with S207.

For ERG11, the complexes with compounds **12**, **14** and **15** show a binding pattern different from that predicted for **16**. Interestingly, in these complexes, the amide group orients toward G309 and T313, which could explain the improved electrostatic energetic component relative to compound **16**. The large Van der Waals contribution to the free energy of binding for compound **16** partially compensates for this gap in the electrostatic interactions, despite compound **12** being predicted to have the lowest free energy of binding to ERG11. Finally, binding to ERG13 also seems to be driven by Van der Waals interactions, as can be seen from Table S4.

## 3. Discussion

The therapeutic potential of halogenated derivatives as antifungal drugs was demonstrated in the present study via inhibitory action on fungal strains which are associated to human diseases. As shown, amides **2** and **14** were the most active compounds against the growth assay of *C. parapsilosis*, with an MIC of 31.25 µg/mL. This pattern of substituents in the aromatic ring possibly contributes to the greater antifungal potency against this species. The amides **12** and **16** were bioactive against *C. paropsilosis*, with an MIC of 125 µg/mL. However, considering an MIC of 250 µg/mL, the two amides with *para* substituents, amides **9** and **15**, had weak inhibitory action on the same strain.

The amide **16** stood out with the smallest MIC (7.8 µg/mL) against *C. krusei* ATCC 14243. This compound, with a bulky alkyl disubstitution in the *meta* position and a hydroxyl group in the *para* position, results in an effective inhibitor of fungal growth, even better than fluconazole, a drug used as standard. Compound **16** also showed bioactivity against eight different *Candida* strains with MICs ranging from 85.3–341.3 µg/mL. In fact, among these fungi used, five are clinical strains resistant to fluconazole. Interestingly, the MIC value against *C. krusei* ATCC 6258 (85.3 mcg/mL) was higher than that determined against *C. krusei* ATCC 14243 (7.8 mcg/mL). This strain was more sensitive to the inhibitory action of compound **16**.

A new multi-resistant yeast, *Candida auris*, appeared in 2009 in East Asia and quickly spread around the world, being classified as a serious threat to public health due to infections with high transmissibility and mortality. Recently, epidemiological reports of *C. auris* outbreaks were released in the context of the COVID-19 pandemic [44], another very serious public health problem in the world [45]. In the present study, compound **16** showed antifungal activity against *C. auris* 01256P CDC, with an MIC of 85.3 µg/mL. Therefore, this compound can be used as a prototype to obtain synthetic analogues with a better antifungal profile against *C. auris*.

Amide **14** was the second most potent compound in the growth assay of *C. krusei*, with an MIC of 31.25 µg/mL, similar to the inhibition concentration achieved against *C. parapsilosis*. This amide was the substance that presented the highest inhibitory potency against both species of fungi, therefore having the best spectrum of antifungal action. Compounds **2**, **9**, **12** and **15** were also bioactive against both species, but at higher concentrations.

A triple substitution on the aromatic ring, one of them being a hydroxyl group in the *para* position and two lipophilic substituents in the *meta* position, is a hypothetical model for the antifungal activity of the synthetized amides against the tested yeasts. This pattern of activity is demonstrated by the inhibitory action of the trisubstituted compounds on the aromatic ring, for example, amides **9**, **14** and **16**. Compound **11** is trisubstituted, but with methoxyl at the *para* position instead of hydroxyl, and it was inactive. Interestingly, amide **14** was more potent than its analogue **9**, suggesting that the presence of the spacer between the carbonyl group and the trisubstituted aromatic ring results in lower inhibitory potency against both *Candida* species. These findings agree with literature reports, such as the activity of syringic acid (MIC = 50 µg/mL) against *Candida albicans* [46], the precursor of derivative **14** (MIC of 31.25 µg/mL), and other benzamides also bioactive against species of this genus of fungi [40].

Furthermore, according to the classification scheme established by Alves et al. [47] for the antifungal potency of synthetic molecules against *Candida* species, MIC < 3.515 μg/mL indicates very strong bioactivity; 3.516–25 μg/mL strong bioactivity; 26–100 μg/mL moderate bioactivity; 101–500 μg/mL weak bioactivity; 500–2000 μg/mL very weak bioactivity and >2000 μg/mL no bioactivity. Compounds **2**, **9**, **12**, **14**, **15** and **16** showed a bioactivity profile ranging from weak to strong. Compound **16** showed weak bioactivity against *C. parapsilosis* and strong bioactivity against *C. krusei* ATCC 14243, while compound **14** exhibited moderate activity against both *Candida* species.

The modeling studies performed with compound **16** point to a multi-target mechanism of action. According to our calculations, the most probable targets of compound **16** in *C. krusei* relate to the redox balance in the cell (ALDH1), signaling pathways (MAPK3) and protein synthesis (PPCTI2). Among the proteins related to ergosterol biosynthesis, ERG13, ERG11 and ERG9 are also predicted as potential binders of this compound. Notably, the multi-target mechanism of action of compound **16** is supported by the predictions made for compounds **12**, **14** and **15**. Among these, **16** is the only one predicted with favorable free energies of binding to all targets.

Regarding the antifungal activity of derivative **16** against *C. parapsilosis*, the proposed targets of the compounds are highly conserved relative to *C. krusei*. Specifically, the residues directly interacting with the modeled ligand are conserved in both species, except for the F139G and Y140F mutations in ALDH1 and ERG11, respectively. Despite the high sequence conservation suggesting the potential inhibition of the targets from both species, this remains to be determined in future works.

When analyzing the obtained results, it must be considered that the biological assays performed focus on the antifungal activity. This is a complex endpoint that should not be solely correlated with enzymes’ inhibition. Other factors such as diverse detoxification mechanisms and membrane permeability to the compounds can lead to differential susceptibility to antifungal agents.

Aldehyde dehydrogenases, such as ALDH1 and ALDH4, are present in a wide range of species and relate to the cellular response to oxidative stress in the cell [48]. Thus, the predicted interaction of compound **16** with these targets might lead to a redox imbalance in the fungus. PPCTI2 belongs to the peptidyl prolyl cis-trans isomerases family of proteins, catalyzing the isomerization of the peptide bond preceding prolines within protein substrates [49]. Despite this family of proteins having not been fully characterized in *Candida* species, some of its members have been proposed as targets capable of overcoming drug resistance in *C. albicans* [50,51].

The three selected potential targets related to ergosterol synthesis are essential for the growth and virulence of *C. albicans* [52], and this particular pathway has been widely exploited for drug development. Finally, MAPK3 (Hog1 mitogen-activated protein kinase) has been extensively investigated in *C. albicans* and proposed by different researchers as an attractive target for the development of new antifungal agents [53,54,55]. Interestingly, this protein kinase has been established as essential for the response to oxidative stress in *C. albicans* [56]. We hypothesize that the simultaneous binding of compound **16** to aldehyde dehydrogenase enzymes and to MAPK3 could lead to a synergic effect compromising the ability of the microorganism to respond to oxidative stress.

Most information reported in the literature establishes *C. albicans* as the model organism to study the mechanism of action of antifungal drugs in *Candida* species. Hence, the proposed mechanism of action of compound **16** in *C. krusei* ATCC 14243 can only be supposed based on the available information. Nevertheless, we consider that the modeling studies performed in this research can guide future experimental efforts toward the prioritization of experiments for the clarification of the mechanism of action of the assayed amide derivatives.

## 4. Materials and Methods

### 4.1. General Information

Purification of the compounds was performed by column chromatography on silica gel 60 (ART. 7734 Merck, St. Louis, MO, USA) using a hexane: ethyl acetate (Hex:EtOAc) solvent gradient and confirmed by analytical thin-layer chromatography on silica gel 60 F_254_, using ultraviolet light at two wavelengths (254 and 366 nm) from a Mineralight apparatus (UVP, Upland, CA, USA) or H_2_SO_4_ in 5% ethanol for detection. FTIR spectra were recorded in a Prestige-21 FTIR spectrometer (Shimadzu, Kyoto, Japan) using KBr pellets. ^1^H- and ^13^C-nuclear magnetic resonance (NMR) spectra were obtained on a MERCURY machine (200 and 50 MHz for ^1^H and ^13^C, respectively). Varian (Palo Alto, CA, USA) in deuterated solvents (CDCl_3_, MeOD or DMSO-*_d6_*) and tetramethylsilane (TMS) was used for the internal standard. Chemical shifts were measured in parts per million (ppm) and coupling constants (*J*) in Hz. Measurements of atomic mass for the compounds was carried out using an Ultraflex II TOF/TOF mass spectrometer (Bruker Daltonik GmbH, Bremen, Germany) equipped with a high-performance solid-state laser (λ = 355 nm) and reflector. The system was operated by the Bruker Daltonik FlexControl 2.4 software package (Bruker, Bremen, Germany). All spectroscopic data are available [35].

### 4.2. General Preparation of N-(4-Halobenzyl)amides

**Figure 5:**
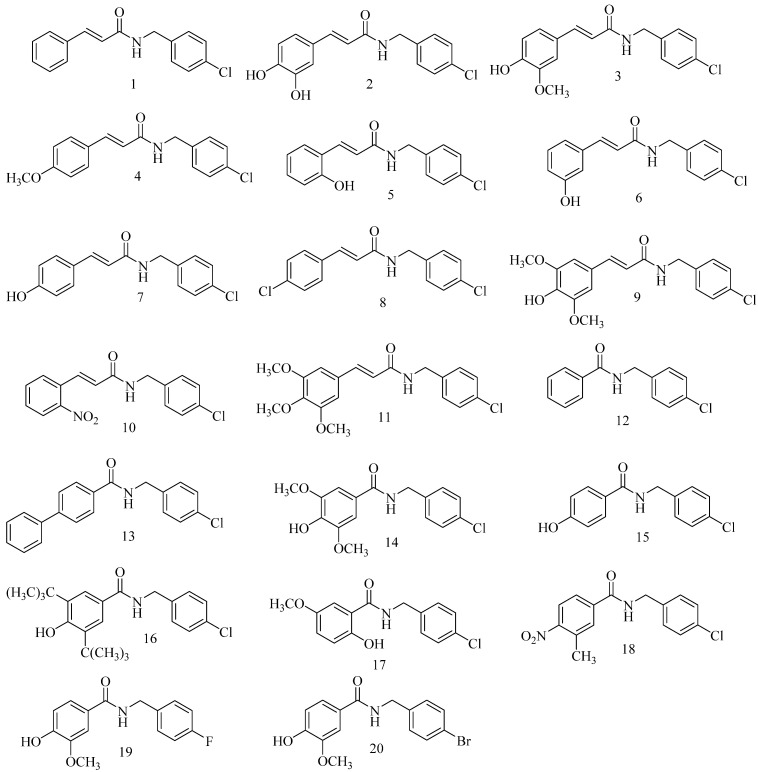
Halogenated amides synthetized via BOP.

The compounds **1**–**20** (Figure 5) were prepared by this procedure: in a 100 mL flask equipped with magnetic stirring, the organic acid (1.35 mmol, 200 mg) was dissolved in dimethylformamide (DMF, 2.7 mL) and trimethylamine (0.14 mL, 1.35 mmol). The solution was cooled in an ice bath (0 °C). Then, 4-halobenzylamine (1.35 mmol) was added. Soon after, a solution of BOP (1.35 mmol) in CH_2_Cl_2_ (10 mL) was added to the flask. The reaction was stirred at 0 °C for 30 min and then for an additional period at room temperature for 2 h. After the reaction, the CH_2_Cl_2_ was removed under reduced pressure, and the solution was poured into a separatory funnel containing water (10 mL) and EtOAc (10 mL). The product was extracted with EtOAc (3 × 10 mL). The organic phase was washed sequentially with 1 N HCl, water, 1 M NaHCO_3_ and water (10 mL of each); it was then dried with Na_2_SO_4_, filtered and concentrated in a rotary evaporator. The amide was purified by gel chromatography on a silica gel column using an EtOAc:Hex mixture gradient of increasing polarity as the mobile phase [21,35].

### 4.3. Evaluation of Antimicrobial Activity

#### 4.3.1. Microbiological Strains and Culture Medium

The microorganisms used in the microbiological tests were strains of *Candida parapsilosis* (ATCC 22019) and *Candida krusei* (ATCC 14243). The culture medium used in the antifungal activity assays was Sabouraud agar dextrose prepared from an initial inoculum suspension according to the 0.5 McFarland scale. Medium was prepared according to the manufacturer’s instructions.

#### 4.3.2. Determination of the Minimum Inhibitory Concentration (MIC)

The broth microdilution (BMD) antifungal susceptibility test was performed according to M27-A3 protocol using RPMI broth (pH 7.0) buffered with 0.165 M MOPS [3-(*N*-morpholino)propanesulfonic acid (Sigma-Aldrich, St Louis, MO, USA) [57]. Compounds were dissolved in dimethyl sulfoxide (DMSO; Sigma-Aldrich) and tested at concentrations ranging from 1.95 to 1000 μg/mL. The yeasts and compounds were incubated in 96-well culture plates at 35 °C for 24 h, and the results were examined visually. The minimum inhibitory concentration (MIC) of each compound was determined as the concentration that inhibited 50% of fungal growth [58].

### 4.4. Evaluation of Antimicrobial Activity Compound **16**

#### 4.4.1. Microbiological Strains and Culture Medium

Eight strains were used for this test: Candida parapsilosis ATCC 22019, C. krusei ATCC 6258, C. auris 01256P derived from CDC B11903 and five clinical strains resistant to fluconazole, including two *C. albicans*, one *C. parapsilosis*, one *C. tropicalis* and one *C. glabrata*. All microorganisms used belong to the Laboratory of Bioprospection in Antimicrobial Molecules (LABIMAN) from Federal University of Ceará. These were seeded on Sabouraud dextrose agar and incubated at 35 °C for 24 h.

#### 4.4.2. Determination of the Minimum Inhibitory Concentration (MIC)

The broth microdilution technique was performed according to document M27-A3 (CLSI, 2008) using RPMI 1640 culture medium (pH 7.0 ± 0.1) buffered with 0.165 M morpholinopropanesulfonic acid (MOPS) (Sigma, St. Louis, MO, USA).

From a 24-h cultivation of the yeasts, carried out in potato agar added with chloramphenicol, an initial inoculum suspension was prepared according to the 0.5 McFarland scale. Then, dilutions were made in RPMI 1640 medium to obtain a final inoculum containing 0.5 to 2.5 × 10³ CFU/mL, which was added to the plate. The microplates containing the inoculum and the drug were incubated at 35 °C (±2 °C) for 24 h. Readings were taken visually. Compound **16** was tested in concentrations ranging from 1024 to 2 µg/mL.

### 4.5. Computational Methods

#### 4.5.1. Molecular Modeling

Molecular modeling was based on our previous studies focusing on the proposal of potential mechanisms of action for bioactive compounds [33,59,60]. Potential targets of compound **16** in *C. krusei* were identified by combining computational target fishing and previous bioactivity data obtained for similar compounds against *C. guilliermondii* [40]. Specifically, in [40], it was reported that this type of compound interferes with the synthesis of ergosterol in *C. guilliermondii*. Afterward, molecular docking of compound **16** was performed against all its potential targets in *C. krusei*. Next, molecular dynamics (MD) simulations were performed for the predicted complexes, and the free energies of binding were computed by means of the molecular mechanics combined with the Poisson–Boltzmann and surface area (MM-PBSA) method. The most probable targets of compound **16** in *C. krusei* were finally proposed according to the results of the estimated free energies of binding.

#### 4.5.2. Targets Selection

Compound **16** was used as query on the Similarity Ensemble Approach (SEA) web server [61] for the identification of its possible targets. Computational target fishing approaches rely on the ligand–receptor interaction data available on different databases, with this data being biased against human proteins. Because of this, a homology-based approach was implemented to identify proteins in *C. krusei* homologs to those identified during the target fishing stage. For this, the sequences of the identified targets were subject to a BLAST [62] search against the *C. krusei* (taxid 4909) proteins in the Reference Sequence proteins (refseq_protein) database using the NCBI BLAST web server implementation (https://blast.ncbi.nlm.nih.gov/, accessed on 4 June 2020). *C. krusei* proteins covered on at least 70% of its length by the BLAST alignment and identical to the query sequence on at least 40% were selected as potential targets of compound **16**.

In addition, some amides derived from vanillic acid highly similar to the compounds studied herein have been demonstrated to interfere with the synthesis of ergosterol in *C. guilliermondii* [40]. Despite the fact that no protein related to ergosterol synthesis was identified in the above step as a potential target for compound **16**, the enzymes involved in this metabolic pathway were included in our investigation. These were defined from the *Candida* Genome Database [63] (Pathway PWY3B3-3). Considering that the metabolic pathways in this database are defined for *C.* *albicans*, the homologous proteins were identified in *C. krusei* through a BLAST search.

#### 4.5.3. Molecular Docking

The molecular docking protocol applied in this research has been described elsewhere [60,64]. In brief, the initial three-dimensional structure of compound **16** was generated with OpenEye’s Omega [65,66], and am1-bcc partial atomic charges were added to it with Molcharge [67]. Since the structures of the investigated proteins from *C. krusei* are unavailable, homology models were generated for them with the SWISS-MODEL server [68].

Docking calculations were performed with the GOLD software [69]. The receptors’ binding sites were defined from the ligands present on the templates used for the homology modeling. Primary docking took place with the ChemPLP scoring function, and 30 different poses of compound **16** were explored for each target with the search efficiency parameter set to 200%. Additionally, the side chains of the residues pointing toward the binding cavity were set as flexible. The predicted binding poses were rescored with the GoldScore, ChemScore and ASP scoring functions of GOLD. A consensus scoring approach was applied to select the most probable binding modes of the ligand to the explored receptors, as described in our previous publications [60,64].

#### 4.5.4. Molecular Dynamics Simulations and Prediction of the Free Energies of Binding

The Amber 20 [70] package was used for MD simulations and the estimation of the free energies of binding. The gaff2 and ff19SB force fields were employed to parametrize the ligand and proteins, respectively. Zn^2+^ ions were parametrized according to the cationic dummy atom model proposed by [71]. The parameters for the NAD and Heme cofactors were taken from the contributed files provided with the Amber 20 distribution [72,73]. All the simulations were run in explicit solvent, following the same energy minimization, heating, equilibration and production steps for all systems.

Complexes (receptor + ligand) were enclosed in truncated octahedron boxes, solvated with OPC water molecules and neutralized with either Na^+^ or Cl^−^ counterions. The solvated systems were sequentially energy-minimized, heated from 0 K to 300 K and equilibrated as previously described [60,64]. Every equilibrated system was used as input to five production runs of 4-ns length each. The initial velocities were randomly initialized before each production run. This procedure yielded a total of 20 ns simulation time per complex.

The free energies of binding of compound **16** to its potential targets were computed using the MM-PBSA method as implemented in the MM-PBSA.py script of Amber 20. Calculations took place across 100 MD snapshots evenly extracted from the five production runs. Snapshots extraction started at 1 ns and continued until the last one for each production run. The ionic strength for MM-PBSA calculations was set to 100 mM.

## 5. Conclusions

The twenty evaluated derivatives resulted in the identification of various antifungal substances with promising MIC values (341.3 to 7.8 µg/mL). Among the bioactive compounds (**2**, **9**, **12**, **14**, **15** and **16**), amide **16** was the most potent one against *Candida krusei* ATCC 14243, with better antifungal activity than the standard drug fluconazole. Comparative analysis of the bioactive compounds suggests that trisubstituted amides with the oxyalkyl or bulk alkyl substituents in *meta* positions and a hydroxyl group in the *para* position have better antifungal activity against *Candida* strains. A computational investigation of the potential mechanism of action of compound **16** in *C. krusei* revealed a possible multi-target mechanism of action involving different cellular processes. These are mainly related to the redox balance in the cell, kinase signaling pathways, protein synthesis and ergosterol synthesis. The data of the present study reveal potent bioactive substances with potential applications in the development of new candidates for antifungal drugs.

## Figures and Tables

**Table 1:** Evaluation of MIC (µg/mL) of the amides derived from cinnamic and benzoic acids in the broth microdilution assay.

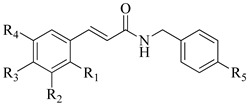 1–11						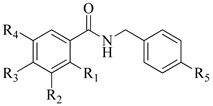 12–20	
Compounds	R_1_	R_2_	R_3_	R_4_	R_5_	MIC ^1^	
						*C. parapsilosis* (ATCC 22019)	*C. krusei* (ATCC 14243)
1	-	-	-	-	Cl	+	+
2	-	OH	OH	-	Cl	31.25	250
3	-	OCH_3_	OH	-	Cl	+	+
4	-	-	OCH_3_	-	Cl	+	+
5	OH	-	-	-	Cl	+	+
6	-	OH	-	-	Cl	+	+
7	-	-	OH	-	Cl	+	+
8	-	-	Cl	-	Cl	+	+
9	-	OCH_3_	OH	OCH_3_	Cl	250	150
10	NO_2_	-	-	-	Cl	+	+
11	-	OCH_3_	OCH_3_	OCH_3_	Cl	+	+
12	-	-	-	-	Cl	125	250
13	-	-	C_6_H_5_	-	Cl	+	+
14	-	OCH_3_	OH	OCH_3_	Cl	31.25	31.25
15	-	-	OH	-	Cl	250	250
16	-	C(CH_3_)_3_	OH	C(CH_3_)_3_	Cl	125	7.8
17	-	OH	-	OCH_3_	Cl	+	+
18	-	CH_3_	-	NO_2_	Cl	+	+
19	-	OCH_3_	OH	-	F	+	+
20	-	OCH_3_	OH	-	Br	+	+
Fluconazole	-	-	-	-		2	16
Voriconazole	-	-	-	-		0.125	0.25

+ Growth of the microorganism; ^1^ MIC was defined in µg/mL and was considered the lowest concentration that produced 50% reduction in fungal cell growth after 24 h of incubation.

**Table 2:** Minimum inhibitory concentrations (MICs) of compound **16** against *Candida* spp.

MIC^50^	
Strains ^a^	Compound 16 ^b^
*C. parapsilosis* ATCC 22019	106.6 µg/mL
*C. krusei* ATCC 6258	85.3 µg/mL
*C. auris* 01256P CDC	85.3 µg/mL
*C. albicans 1*	341.3 µg/mL
*C. albicans 2*	128 µg/mL
*C. tropicalis*	106.6 µg/mL
*C. parapsilosis*	128 µg/mL
*C. glabrata*	128 µg/mL

^a^ Isolated collection yeast strains; ^b^ MIC was defined as the lowest concentration that produced 50% reduction in fungal cell growth after 24 h. Note: the DMSO concentration in the well referring to 1024 µg/mL was below 2.5%, showing no activity and no interference in the tests.

**Table 3:** List of the 37 potential targets of compound **16** in *C. krusei*.

UniProt Accession	ID ^a^	Description
A0A099P395 ^b^	ALDH5	Aldehyde dehydrogenase 5, mitochondrial
A0A2U9R6B5 ^b^	ALDH1	Aldedh domain-containing protein
A0A099P647 ^b^	ALDH3	Aldedh domain-containing protein
A0A2U9R5Y1 ^b^	ALDH2	Aldedh domain-containing protein
A0A2U9R723 ^b^	ALDH4	Aldedh domain-containing protein
A0A099P843 ^b^	MAPK1	Mitogen-activated protein kinase CEK1
A0A099NVZ1 ^b^	MAPK2	Mitogen-activated protein kinase
A0A1V2LG11 ^b^	MAPK3	Mitogen-activated protein kinase;uncharacterized protein HOG1
A0A1Z8JVT5 ^b^	PK1	Protein kinase domain-containing protein
A0A2U9R7Y2 ^b^	PK2	Protein kinase domain-containing protein
A0A099P0U0 ^b^	HDA1	Histone deacetylase RPD3
A0A2U9RAK0 ^b^	HDA2	Histone deacetylase
A0A1V2LMB8 ^b^	PPCTI-D	Peptidyl-prolyl cis-trans isomerase D
A0A2U9QXT4 ^b^	PPCTI1	Peptidyl-prolyl cis-trans isomerase
A0A099P2W3 ^b^	PPCTI2	Peptidyl-prolyl cis-trans isomerase
A0A099P773 ^b^	PPCTI3	Peptidyl-prolyl cis-trans isomerase
A0A1Z8JS57 ^b^	CA	Carbonic anhydrase
A0A2U9RBI7 ^c^	IDI1	Isopentenyl-diphosphate Delta-isomerase
A0A1Z8JJU5 ^c^	MVD	Diphosphomevalonate decarboxylase
A0A2U9R9Y8 ^c,d^	ERG8	Phosphomevalonate kinase
A0A2U9R5L9 ^c^	ERG12	Mevalonate kinase
A0A2U9R6J4 ^c^	HMG1	3-hydroxy-3-methylglutaryl coenzyme A reductase
A0A099P154 ^c^	ERG13	3-hydroxy-3-methylglutaryl coenzyme A synthase
A0A099P5C0 ^c^	ERG10	Acetyl-CoA C-acetyltransferase IA
A0A099P078 ^c,d^	ERG26	Sterol-4-alpha-carboxylate 3-dehydrogenase
A0A1Z8JS11 ^c,d^	ERG27	3-keto-steroid reductase
A0A2U9RAJ8 ^c,d^	ERG4	Delta(24(24(1)))-sterol reductase
A0A099P3X9 ^c,d^	ERG5	C-22 sterol desaturase
A0A099P0V3 ^c,d^	ERG3	C-5 sterol desaturase
A0A2U9QYN8 ^c^	ERG2	C-8 sterol isomerase
A0A1V2LUI2 ^c^	ERG6	Sterol 24-C-methyltransferase
A0A099NZM6 ^c,d^	ERG25	Methylsterol monooxygenase
A0A099P4I0 ^c,d^	ERG24	Delta(14)-sterol reductase
A9YUC7 ^c^	ERG11	Lanosterol 14-alpha demethylase
A0A2U9R113 ^c^	ERG9	Squalene synthase
A0A2U9R171 ^c^	ERG7	Lanosterol synthase
A0A099NX62 ^c^	ERG1	Squalene monooxygenase
A0A099P5M1 ^c^	ERG20	(2E,6E)-farnesyl diphosphate synthase

^a^ ID used along the manuscript for each target; ^b^ Identified by the target fishing approach; ^c^ Part of the ergosterol synthesis pathway; ^d^ Homology model discarded due to low quality.

## Data Availability

The data presented in this study are available in the article and associated supplementary material and in https://repositorio.ufpb.br/jspui/handle/tede/8806.

## Supplementary Materials

The following are available online at https://www.mdpi.com/article/10.3390/ijms23010419/s1.
