# Supplementary material for: Antifungal Activity of N-(4-Halobenzyl)amides against Candida spp. and Molecular Modeling Studies
Source: Int J Mol Sci. 2021 Dec 31;23(1):419. doi: 10.3390/ijms23010419 (PMC8745543; doi:10.3390/ijms23010419)
Supplement: Supplementary file 1 [file ijms-23-00419-s001.zip › ijms-1434761-supplementary.pdf]

Supplementary Materials for Article

Antifungal Activity of *N*-(4-Halobenzyl)amides Against *Candida parapsilosis* and *Candida krusei* and Molecular Modeling Studies

Yunierkis Perez-Castillo[1], Ricardo Carneiro Montes[2], Cecília Rocha da Silva[3], João Batista de Andrade Neto[3], Celidarque da Silva Dias[2], Hélio Vitoriano Nobre Júnior[3], Damião Pergentino de Sousa[2]*

[1]Bio-Cheminformatics Research Group and Escuela de Ciencias Físicas y Matemáticas, Universidad de Las Américas, Quito 170504, Ecuador

[2] Department of Pharmaceutical Sciences, Federal University of Paraíba, 58051-900 João Pessoa, Paraíba, Brazil;

[3] Laboratory of Bioprospection in Antimicrobial Molecules (LABIMAN), Drug Research and Development Center (NPDM), Federal University of Ceara, 60020-181, Fortaleza, CE, Brazil;

*For Correspondence: damiao_desousa@yahoo.com.br; Tel.: +55-83-3216-7502

Table S1. Summary of the homology modeling stage.

ID	PDB Template	Coverage[(a)]	QMEAN[(b)]
ALDH5	4qf6	0.9	0.02
ALDH1	6alj	0.9	-0.2
ALDH3	6dum	0.93	-1.01
ALDH2	4qet	0.95	-0.63
ALDH4	5ac2	0.95	-1.2
MAPK1	2b9h	0.97	-1.62
MAPK2	4zzm	0.95	-1.85
MAPK3	3mh3	0.9	-1.29
PK1	5z33	0.82	-1.71
PK2	1stc	0.67	-1.63
HDA1	7kbg	0.81	0.34
HDA2	4a69	0.8	-0.28
PPCTI-D	1iip	0.93	0.63
PPCTI1	1xo7	0.78	1.7
PPCTI2	3o7t	0.99	2.19
PPCTI3	5yb9	0.9	2.05
CA	6gwu	0.8	-0.65

IDI1	2dho	0.8	-1.37
MVD	6xr5	0.98	-1.66
ERG12	2r3v	0.88	-2.8
HMG1	1hw8	0.41	-0.52
ERG13	2p8u	0.99	-1.69
ERG10	6aqp	0.94	-0.59
ERG2	5hk1	0.56	-2.87
ERG6	6uv6	0.54	-1.79
ERG11	5esj	0.98	-0.7
ERG9	3vja	0.8	-2.24
ERG7	1w6k	0.98	-1.88
ERG1	6c6n	0.9	-2.21
ERG20	4rxc	0.99	-1.02
ERG8[(c)]	3gon	0.66	-5.78
ERG26[(c)]	6q94	0.92	-4.13
ERG4[(c)]	4quv	0.83	-5.7
ERG5[(c)]	5veu	0.85	-4.84
ERG3[(c)]	4zr0	0.39	-5.19
ERG25[(c)]	4zr1	0.45	-4.9
ERG24[(c)]	4quv	0.94	-4.93

[(a)] Coverage of the query sequence by the template, [(b)] Swiss-Model QMEAN score [(c)] Models discarded due to low quality

Table S2 Results of docking compound 16 to its 29 potential targets in C. Krusei.

Target	Pose	PLP[(a)]	Z_PLP[(b)]	GS[(c)]	Z_GS[(d)]	CS[(e)]	Z_CS[(f)]	ASP[(g)]	Z_ASP[(h)]	Aggregated Z-Score
ALDH1	1	59.08	1.99	18.59	0.69	16.18	1.97	24.17	2.2	1.71
	2	52.29	1.28	13.67	0.45	15.41	1.85	20.61	1.73	1.33
	3	63.21	2.43	24.11	0.95	6.99	0.59	15.16	1	1.24
	4	51.68	1.21	20.32	0.77	10.58	1.13	15.93	1.11	1.05
ALDH2	1	64.19	1.48	30.89	0.96	25.28	1.02	36.68	1.3	1.19
	2	65.18	1.57	21.83	0.58	23.13	0.77	35.65	1.2	1.03
ALDH3	1	67.89	0.9	34.85	1.88	25.3	1.61	34.53	1.62	1.5
	2	78.85	2.47	1.54	-0.27	23.71	1.09	36.62	2.07	1.34
ALDH4	1	84.31	2.55	30.74	0.83	32.49	1.08	45.1	2.81	1.82
	2	79.26	1.66	26.6	0.62	33.29	1.33	40.79	1.63	1.31
	3	79.08	1.63	23.97	0.49	32.97	1.23	40.17	1.46	1.2
	4	78.25	1.48	22.18	0.4	34.75	1.79	37.28	0.67	1.09
ALDH5	1	61.61	1.36	23.7	1.18	20.48	0.74	29.32	1.42	1.18
	2	65.21	1.75	26.97	1.4	20.57	0.75	21.38	0.44	1.09
	3	57.41	0.9	25.56	1.31	23.98	1.29	24.24	0.79	1.07
CA	1	49.82	1.87	8.21	0.51	14.37	1.43	18.43	1.63	1.36
	2	47.47	1.52	6.76	0.38	15.09	1.56	18.38	1.63	1.27
	3	47.02	1.45	7.34	0.44	14.65	1.48	19.05	1.72	1.27
HDA1	1	49.82	1.87	8.21	0.51	14.37	1.43	18.43	1.63	1.36
	2	47.47	1.52	6.76	0.38	15.09	1.56	18.38	1.63	1.27
	3	47.02	1.45	7.34	0.44	14.65	1.48	19.05	1.72	1.27
HDA2	1	71.78	1.8	15.11	0.69	26.84	1.54	32.55	1.36	1.35
	2	73.33	1.99	14.25	0.64	21.14	0.25	31.78	1.22	1.03
	3	67.65	1.28	6.64	0.18	25.59	1.26	32.35	1.32	1.01
MAPK1	1	58.1	0.42	16.77	0.69	24.09	1.88	21.13	1.39	1.1
MAPK2	1	59.98	2.05	13.97	1.12	19.67	1.04	16.25	0.22	1.11
MAPK3	1	77.18	2.44	12.88	0.21	29.44	1.41	29.76	1.91	1.49
	2	69.05	1.02	20.97	0.61	30.08	1.57	28.92	1.7	1.22
	3	70.7	1.31	18.78	0.5	27.47	0.92	28.24	1.52	1.06
PK1	1	55.21	0.52	15.2	0.71	26.35	2.22	23.47	1.74	1.3
	2	59.39	1.31	8.6	0.32	22.92	1.23	22.92	1.57	1.11
	3	60.33	1.48	11.22	0.48	23.41	1.37	20.78	0.9	1.06
PK2	1	69.19	0.94	14.83	0.6	30.77	2.19	28.69	1.5	1.31
	2	73.08	1.72	10.11	0.28	24.61	0.34	30.38	1.97	1.08
PPCTI1	1	61.99	2.24	14.22	0.23	18.8	0.86	30.27	2.15	1.37
	2	58.32	1.67	-7.8	0.15	22.95	1.78	27.7	1.6	1.3
PPCTI2	1	53.3	1.63	13.11	0.77	18.74	1.71	25.25	2.09	1.55
	2	52.29	1.47	11.01	0.66	18.16	1.56	23.86	1.72	1.35
	3	55.12	1.92	16.08	0.92	12.36	0.1	22.13	1.26	1.05
PPCTI3	1	56.86	2.01	16.56	1.06	16.09	0.73	28.14	1.8	1.4
PPCTI-D	1	57.4	3.23	-1.56	-0.08	10.8	0.64	18.57	1.17	1.24
	2	46.94	1.34	10.45	1.21	12.25	1.22	16.71	0.67	1.11
	3	45.46	1.07	8.67	1.02	14.01	1.93	15.28	0.29	1.08

ERG10	1	66.81	2.65	27.83	2.03	24.46	1.43	28.65	1.93	2.01
	2	62.5	1.83	8.59	0.6	26.42	2.19	27.32	1.51	1.53
ERG11	1	73.16	1.94	24.8	0.84	31.54	1.33	24.37	0.44	1.14
	2	79.87	2.88	22.48	0.71	23.09	-0.01	26.26	0.71	1.07
	3	63.22	0.55	17.41	0.41	30.02	1.09	35.07	2.01	1.01
ERG12	1	48.96	1.06	-7.97	-0.58	18.99	2.12	18.14	1.81	1.1
ERG13	1	56.96	2.78	12.37	0.97	21.84	1.5	23.79	2.49	1.93
ERG20	1	60.01	2.22	21.35	1.56	13.02	0.67	21.44	1.53	1.49
	2	56.1	1.68	11.09	0.83	16.15	1.57	18.48	0.91	1.25
	3	51.95	1.11	9.76	0.74	16.1	1.56	21.64	1.57	1.25
ERG2	1	58.17	0.96	13.18	0.51	25.98	1.51	31.94	1.76	1.19
ERG6	1	56.08	1.28	20.97	0.24	18.04	1.5	31.76	1.74	1.19
	2	56.93	1.46	1.89	0.17	17.75	1.44	31.24	1.65	1.18
ERG7	1	71.65	1.84	26.46	0.51	22.79	1.26	36.36	1.72	1.33
	2	61.8	0.8	23.63	0.4	24.55	1.53	34.98	1.52	1.06
ERG9	1	64.08	1.31	6.41	0.31	27.9	1.64	28.09	1.32	1.14
	2	66.55	1.92	-5.58	-0.55	27.46	1.47	28.64	1.53	1.09
	3	63.28	1.11	7.14	0.36	26.31	1.01	28.94	1.64	1.03
HMG1	1	42.8	2.21	4.62	0.19	6.86	1.38	10.81	1.86	1.41
IDI1	1	59.95	1.99	14.23	0.21	19.44	2.04	23.45	2.41	1.66
	2	59.6	1.91	21.25	0.87	15.78	0.85	18.29	0.91	1.14
	3	54.3	0.79	19.45	0.7	16.91	1.22	21.02	1.7	1.1
MVD	1	40.14	3.72	10.83	1.05	2.99	2.83	-5.45	0.09	1.92
	2	25.67	0.68	1.91	0.74	-1.8	1.17	3.47	2.52	1.27

[a] PLP score, [b] PLP Z-score, [c] GoldScore score, [d] GoldScore Z-score, [e] ChemScore score, [f] ChemScore Z-score, [g] ASP score, [h] ASP Z-score

Table S3. Predicted free energies of binding of compound 16 to its predicted targets.

Target	Conformer	MM-PBSA Component							ΔG Total
		VD WAALS	EEL	EPB	ENPOLAR	EDISPER	ΔG gas	ΔG solv	
ALDH1	1	-45.40	-26.61	48.69	-31.87	55.50	-72.00	72.32	0.32
	2	-49.68	1.93	25.84	-35.04	58.49	-47.75	49.29	1.54
	3	-55.33	-15.58	41.30	-39.94	66.02	-70.91	67.39	-3.52
	4	-57.32	-14.57	37.60	-39.85	67.62	-71.89	65.37	-6.52
ALDH2	1	-49.46	-15.05	43.51	-35.12	60.77	-64.51	69.16	4.65
	2	-43.16	-23.29	43.00	-31.61	55.93	-66.45	67.33	0.87
ALDH3	1	-48.42	-25.39	52.91	-35.11	61.94	-73.81	79.74	5.92
	2	-47.96	-20.47	50.81	-33.23	58.10	-68.43	75.67	7.25
ALDH4	1	-51.57	-8.51	34.73	-35.89	61.25	-60.08	60.08	0.01
	2	-50.25	-30.54	52.18	-36.31	61.64	-80.79	77.51	-3.28
	3	-49.47	-13.04	34.36	-35.04	58.77	-62.51	58.09	-4.41
	4	-49.68	-11.63	35.92	-35.92	60.35	-61.31	60.35	-0.95
ALDH5	1	-49.98	-16.13	40.86	-36.19	61.11	-66.11	65.78	-0.33
	2	-52.93	-8.31	35.36	-37.69	64.48	-61.23	62.15	0.91
	3	-45.40	-17.97	39.26	-32.81	55.72	-63.37	62.17	-1.21
CA	1	-35.86	-2.18	16.10	-25.75	44.49	-38.05	34.84	-3.21
	2	-33.27	-7.22	22.39	-23.92	41.68	-40.49	40.14	-0.35
	3	-37.72	-5.52	22.95	-27.82	46.53	-43.23	41.65	-1.58
HDA1	1	-35.82	-11.41	33.10	-26.93	44.12	-47.23	50.28	3.05
	2	-33.88	-16.02	35.03	-27.42	44.32	-49.89	51.93	2.04
	3	-32.85	-8.99	28.26	-25.74	42.32	-41.85	44.83	2.98
HDA2	1	-35.52	-13.29	34.38	-27.46	45.06	-48.81	51.99	3.17
	2	-33.56	-0.73	22.95	-25.84	43.34	-34.28	40.45	6.17
	3	-39.31	-11.57	34.36	-29.85	48.26	-50.88	52.77	1.89
MAPK1	1	-48.07	-10.36	42.10	-34.05	58.44	-58.43	66.49	8.06
MAPK2	1	-38.83	-2.71	23.38	-27.78	48.13	-41.54	43.73	2.19
MAPK3	1	-53.26	-32.63	58.44	-39.81	65.82	-85.90	84.45	-1.45
	2	-54.51	-30.32	54.26	-39.09	63.63	-84.82	78.80	-6.02
	3	-52.61	-29.81	56.52	-39.63	66.15	-82.42	83.03	0.62
PK1	1	-48.54	-12.57	39.54	-34.73	61.24	-61.11	66.06	4.94
	2	-42.93	-13.77	39.85	-31.43	54.16	-56.70	62.58	5.88
	3	-43.24	-28.96	53.84	-32.83	56.88	-72.20	77.88	5.68
PK2	1	-50.38	-8.94	41.22	-35.92	61.47	-59.31	66.78	7.47
	2	-56.05	-23.72	53.76	-38.88	64.62	-79.77	79.50	-0.27
PPCTI1	1	-35.45	-6.82	23.79	-25.65	43.61	-42.28	41.74	-0.54
	2	-27.50	-10.38	21.61	-21.18	36.09	-37.87	36.52	-1.35
PPCTI2	1	-31.55	-6.33	18.17	-22.62	37.59	-37.88	33.13	-4.75
	2	-28.31	-5.67	17.13	-20.77	35.45	-33.97	31.81	-2.16
	3	-37.50	-4.51	21.01	-25.58	43.91	-42.01	39.33	-2.68
PPCTI3	1	-41.94	-19.16	36.99	-29.25	50.03	-61.09	57.77	-3.32
PPCTI-D	1	-42.88	-12.93	32.12	-29.94	49.24	-55.81	51.42	-4.39
	2	-38.19	-13.78	33.79	-27.36	47.51	-51.98	53.94	1.96

	3	-39.93	-13.52	31.54	-28.25	48.80	-53.45	52.09	-1.36
ERG10	1	-43.38	-23.91	46.41	-32.20	53.16	-67.29	67.37	0.08
	2	-38.79	-10.07	31.77	-29.13	49.73	-48.86	52.38	3.53
	1	-59.47	-2.01	29.89	-39.57	68.63	-61.48	58.95	-2.54
ERG11	2	-53.53	-5.21	33.29	-37.06	65.12	-58.74	61.35	2.62
	3	-54.96	-8.77	33.60	-39.71	68.14	-63.74	62.03	-1.71
ERG12	1	-35.98	-12.01	28.74	-28.93	47.70	-47.99	47.51	-0.48
ERG13	1	-51.33	-13.30	36.64	-35.69	61.00	-64.63	61.95	-2.69
	1	-45.90	-20.28	52.72	-31.86	57.06	-66.19	77.92	11.73
ERG20	2	-45.70	-25.72	54.53	-32.90	57.38	-71.41	79.02	7.60
	3	-45.34	-10.61	42.06	-32.08	56.41	-55.95	66.38	10.43
ERG2	1	-54.61	-15.95	49.75	-38.94	67.30	-70.56	78.11	7.56
ERG6	1	-52.11	-8.28	43.67	-35.21	62.33	-60.39	70.78	10.39
	2	-52.03	-11.73	57.07	-34.57	60.46	-63.76	82.95	19.19
ERG7	1	-57.19	-10.60	49.99	-39.48	68.82	-67.79	79.32	11.53
	2	-58.26	-13.58	53.74	-39.64	69.19	-71.84	83.29	11.44
	1	-49.38	-11.13	35.78	-35.27	59.72	-60.51	60.23	-0.27
ERG9	2	-32.77	-1.42	13.04	-25.09	45.87	-34.19	33.81	-0.38
	3	-41.91	-11.48	28.74	-30.77	53.18	-53.39	51.14	-2.24
HMG1	1	-43.04	-18.67	49.64	-31.67	57.29	-61.70	75.26	13.55
	1	-47.69	-16.97	61.28	-33.50	59.87	-64.65	87.65	22.99
IDI1	2	-48.57	-28.39	62.59	-35.16	59.20	-76.96	86.63	9.67
	3	-41.10	-22.65	58.22	-31.29	53.82	-63.76	80.76	17.00
MVD	1	-50.48	-22.58	54.77	-35.70	62.32	-73.06	81.39	8.33
	2	-45.79	-31.73	61.49	-34.31	59.35	-77.52	86.53	9.01

Table S4. Predicted free energies of binding of compounds 12, 14 and 15 to its predicted targets.

Target	Compound	MM-PBSA Component							ΔG TOTAL
		VD WAALS	EEL	EPB	ENPOLAR	EDISPER	ΔG gas	ΔG solv	
ALDH1	16	-57.32	-14.57	37.60	-39.85	67.62	-71.89	65.37	-6.52
	12	-32.58	-1.88	19.48	-22.73	37.28	-34.46	34.03	-0.43
	14	-46.20	-13.66	37.57	-31.82	53.20	-59.86	58.95	-0.91
	15	-36.97	-27.89	39.11	-27.62	44.81	-64.87	56.30	-8.57
MAPK3	16	-54.51	-30.32	54.26	-39.09	63.63	-84.82	78.80	-6.02
	12	-35.74	-23.10	39.90	-26.42	41.97	-58.84	55.45	-3.39
	14	-42.64	-29.22	58.03	-32.26	53.55	-71.86	79.32	7.46
	15	-37.63	-23.39	51.62	-26.86	44.51	-61.02	69.27	8.25
PPCTI2	16	-31.55	-6.33	18.17	-22.62	37.59	-37.88	33.13	-4.75
	12	-22.92	-7.91	17.64	-17.84	29.16	-30.83	28.95	-1.87
	14	-20.95	-5.19	13.50	-16.02	26.25	-26.15	23.73	-2.42
	15	-17.65	-5.85	14.26	-13.78	22.64	-23.50	23.11	-0.39
ERG13	16	-51.33	-13.30	36.64	-35.69	61.00	-64.63	61.95	-2.69
	12	-32.69	-6.05	27.43	-23.55	40.41	-38.74	44.30	5.56
	14	-40.26	-10.58	30.63	-27.81	46.07	-50.84	48.89	-1.94
	15	-38.88	-14.24	36.24	-26.26	44.70	-53.12	54.68	1.56
ERG11	16	-59.47	-2.01	29.89	-39.57	68.63	-61.48	58.95	-2.54
	12	-38.93	-9.55	26.75	-25.69	43.58	-48.48	44.64	-3.84
	14	-46.05	-7.49	34.53	-30.67	53.78	-53.55	57.63	4.08
	15	-41.07	-9.49	31.83	-26.48	44.52	-50.56	49.87	-0.69
ERG9	16	-41.91	-11.48	28.74	-30.77	53.18	-53.39	51.14	-2.24
	12	-26.76	-3.91	15.26	-20.48	34.93	-30.67	29.71	-0.96
	14	-33.88	-11.54	28.60	-25.38	43.24	-45.41	46.46	1.05
	15	-25.78	-9.07	22.35	-20.64	34.30	-34.85	36.02	1.17
